# *Vibrio cholerae* pathogenicity island 2 encodes two distinct types of restriction systems

**DOI:** 10.1128/jb.00145-24

**Published:** 2024-08-12

**Authors:** Grazia Vizzarro, Alexandre Lemopoulos, David William Adams, Melanie Blokesch

**Affiliations:** 1 Laboratory of Molecular Microbiology, Global Health Institute, School of Life Sciences, Ecole Polytechnique Fédérale de Lausanne (EPFL), Lausanne, Switzerland; University of California San Francisco, San Francisco, California, USA

**Keywords:** *V. cholerae*, pathogenicity island, restriction systems, DNA modification

## Abstract

In response to predation by bacteriophages and invasion by other mobile genetic elements such as plasmids, bacteria have evolved specialized defense systems that are often clustered together on genomic islands. The O1 El Tor strains of *Vibrio cholerae* responsible for the ongoing seventh cholera pandemic (7PET) contain a characteristic set of genomic islands involved in host colonization and disease, many of which contain defense systems. Notably, *Vibrio* pathogenicity island 2 contains several characterized defense systems as well as a putative type I restriction-modification (T1RM) system, which, interestingly, is interrupted by two genes of unknown function. Here, we demonstrate that the T1RM system is active, methylates the host genomes of a representative set of 7PET strains, and identify a specific recognition sequence that targets non-methylated plasmids for restriction. We go on to show that the two genes embedded within the T1RM system encode a novel two-protein modification-dependent restriction system related to the GmrSD family of type IV restriction enzymes. Indeed, we show that this system has potent anti-phage activity against diverse members of the *Tevenvirinae*, a subfamily of bacteriophages with hypermodified genomes. Taken together, these results expand our understanding of how this highly conserved genomic island contributes to the defense of pandemic *V. cholerae* against foreign DNA.

**IMPORTANCE:**

Defense systems are immunity systems that allow bacteria to counter the threat posed by bacteriophages and other mobile genetic elements. Although these systems are numerous and highly diverse, the most common types are restriction enzymes that can specifically recognize and degrade non-self DNA. Here, we show that the *Vibrio* pathogenicity island 2, present in the pathogen *Vibrio cholerae*, encodes two types of restriction systems that use distinct mechanisms to sense non-self DNA. The first system is a classical Type I restriction-modification system, and the second is a novel modification-dependent type IV restriction system that recognizes hypermodified cytosines. Interestingly, these systems are embedded within each other, suggesting that they are complementary to each other by targeting both modified and non-modified phages.

## INTRODUCTION

Mobile genetic elements (MGEs) such as plasmids, transposons, and integrative-conjugative elements can confer significant fitness advantages by facilitating the transfer of beneficial traits to the host bacterium, including key virulence factors or antibiotic-resistant genes (1). However, their maintenance and or activity can also impose a metabolic burden on the host cell, while elements that integrate on the chromosome have the potential to disrupt important genomic features (2). Furthermore, the replication of some MGEs such as lytic bacteriophages (phages) can result in the death of the host cell (3). Indeed, predation by phages, which are ubiquitous bacterial viruses, has imposed a strong evolutionary pressure to develop multiple lines of defense against these MGEs, including a vast array of specialized defense systems (4, 5).

Upon recognizing an infection, these systems can either respond directly by degrading the invading non-self DNA and thus provide individual level protection or alternatively can sacrifice the host cell prior to phage-induced lysis to protect the surrounding population (abortive infection) (6). The most common and best-studied defense systems are restriction-modification (RM) systems, which use restriction enzymes to directly degrade non-self DNA (5). Types I–III RM systems are modification-blocked enzymes that recognize specific DNA sequences and only cut DNA when it is unmodified, while the corresponding sequences in the host genome are protected by epigenetic modification with a cognate methylase (7–9). In contrast, type IV systems are modification-dependent enzymes that can recognize and degrade invading DNA with specific modifications, which are used by some phages to avoid restriction by modification-blocked systems (9, 10).

Diverse defense systems, including RM systems, tend to cluster together within genomic islands known as “defense islands” (11–13). This pattern also applies to the defense systems identified so far in *Vibrio cholerae*, the causative agent of cholera. This bacterium features specialized islands crucial to its pathogenic evolution. Indeed, only certain *V. cholerae* strains, referred to as toxigenic isolates, can cause cholera. This ability is due to the presence of two key virulence/colonization factors: the cholera toxin (CT) and toxin-coregulated pilus, encoded on the CTXΦ prophage and the *Vibrio* pathogenicity island 1, respectively (14–16). The ongoing seventh cholera pandemic is caused by the O1 El Tor *V. cholerae* lineage (7PET), which uniquely carries the *Vibrio* seventh pandemic islands I and II (VSP-I and VSP-II), characteristic of the 7PET strains (17, 18). These genomic islands are implicated in defense, as they encode for instance CBASS and AvcD systems (VSP-I) and the Lamassu system DdmABC on VSP-II (19–23). Additionally, toxigenic *V. cholerae* strains carry the *Vibrio* pathogenicity island 2 (VPI-2), which is believed to enhance pathogenicity by giving the pathogen a competitive advantage in using sialic acid as a carbon source during gut colonization (24–26). This capability is encoded within the island’s *nan-nag* genomic region (24–26). Moreover, the island houses several genes believed to protect against MGEs, including (i) a predicted Zorya type I system, a phage defense system identified across a wide range of bacterial genomes and experimentally studied primarily through *Escherichia coli* homologs (12, 27); (ii) the DNA defense module DdmDE that targets and degrades small multicopy plasmids (23); and (iii) a gene cluster/operon predicted to encode a type I restriction-modification (T1RM) system (24). The presence of both predicted and established defense systems encoded within VPI-2 suggests that it may serve as a genuine defense island.

In this study, we set out to characterize the predicted T1RM operon within VPI-2. We show that the T1RM system promotes methylation of the genomes of 7PET *V. cholerae* strains, and identify a specific recognition sequence that can target non-self-derived plasmids for restriction. Furthermore, we discovered two genes embedded within the T1RM operon that form a novel modification-dependent restriction system related to the GmrSD family of type IV restriction enzymes, which we term TgvAB. When produced in *E. coli*, this system has potent anti-phage activity against phages with hypermodified genomes. Collectively, these findings enhance our understanding of how this highly conserved genomic island contributes to the defense of pandemic *V. cholerae* against foreign DNA.

## RESULTS AND DISCUSSION

### *In silico* analysis of VPI-2 and the T1RM cluster

**Fig 1:**
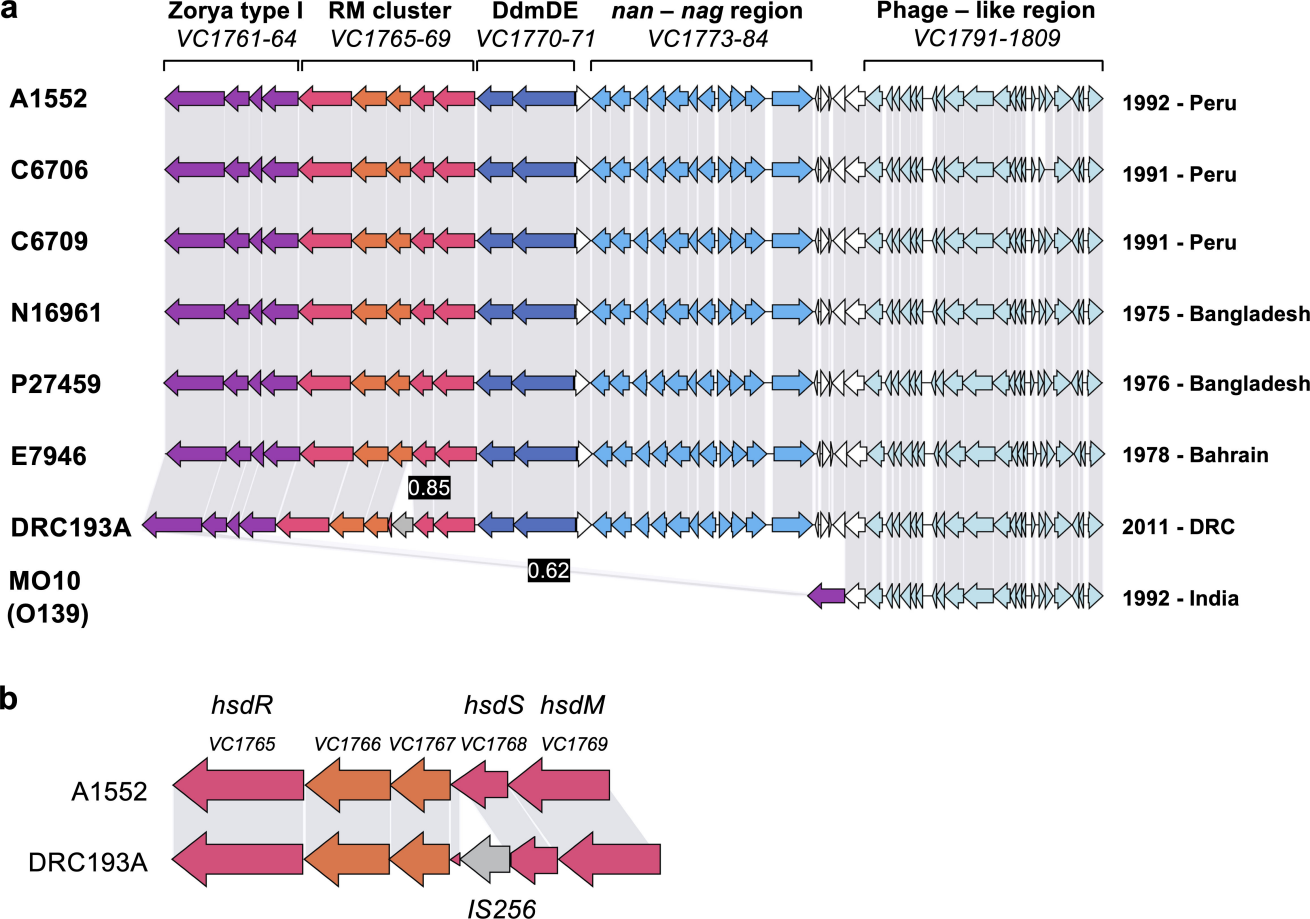
VPI-2 exhibits high conservation across 7PET *V. cholerae* strains. (a) Comparative genome alignment of the VPI-2 across a selection of 7PET O1 and O139 strains, isolated between 1975 and 2011. The genomes of these strains are displayed alongside their designated strain names (left) and their isolation dates and locations (right). Coding sequences within the genomes are represented by arrows, with gray bars connecting them to indicate amino acid identity percentages at or above a threshold of 0.93. Instances of lower identity are highlighted in black boxes. Gene locus tags are derived from the reference genome of strain N16961. Predicted or established functions are labeled above each cluster. (b) Close-up examination of the *VC1765–69* gene cluster in strains A1552 and DRC193A reveals three genes responsible for the components of the putative T1RM system (*hsdR, hsdS, hsdM*). A comparative alignment highlights the disruption of the T1RM cluster in strain DRC193A, caused by an IS*256* transposon insertion within *hsdS*.

Although VPI-2 was discovered over 20 years ago (24), the genes it carries have not yet been fully characterized. To begin bridging this knowledge gap, we started by re-evaluating the gene content of the island. Consistent with earlier findings (24), this revealed that VPI-2 is highly conserved among a set of 7PET O1 strains isolated between 1975 and 2011. Our analysis confirmed the island’s modular structure as described by Jermyn and Boyd (24, 28), including a predicted Zorya system (12) encoded by genes *VC1761–64* [as per reference strain N16961; (29)], a predicted T1RM system (*VC1765–69*) (24), the DdmDE defense module (*VC1770–71)* (23), the *nan-nag* sialic acid utilization cluster (*VC1773–1784*), and a region with phage-like properties (*VC1791–1809*) (24) (Fig. 1a). Notably, O139 serogroup strains such as MO10 carry a highly truncated version of VPI-2 that retains only the phage-like region (24, 30) (Fig. 1a).

Given the observed conservation of VPI-2 and the presence of established defense systems, we explored the possibility that the putative T1RM system was also actively involved in restricting foreign DNA. Interestingly, the previously annotated T1RM region sits within a five-gene operon, of which three genes encode homologs of the known T1RM components (Fig. 1b). These host-specificity determinant (*hsd*) genes encode the specificity subunit HsdS, which recognizes a specific DNA recognition sequence; the methylase subunit HsdM, which methylates (and therefore protects) the recognition sequences in the host genome; and HsdR, the restriction enzyme subunit, which upon encountering foreign DNA with an unmethylated recognition sequence translocates the flanking DNA and cleaves at variable distances from the recognition site (7, 31–33). These components function together as multi-subunit complexes capable of both methylating and restricting DNA. Importantly, restriction requires a pentameric complex of 2HsdR + 2HsdM + HsdS, and although HsdR is dispensable for methylation, HsdS is required for both activities (7, 31). Interestingly, two genes of unknown function are embedded within the T1RM cluster (*VC1767–66*; Fig. 1b), which we characterize in the subsequent sections below.

### Deciphering the recognition motif of VPI-2’s T1RM system

**Fig 2:**
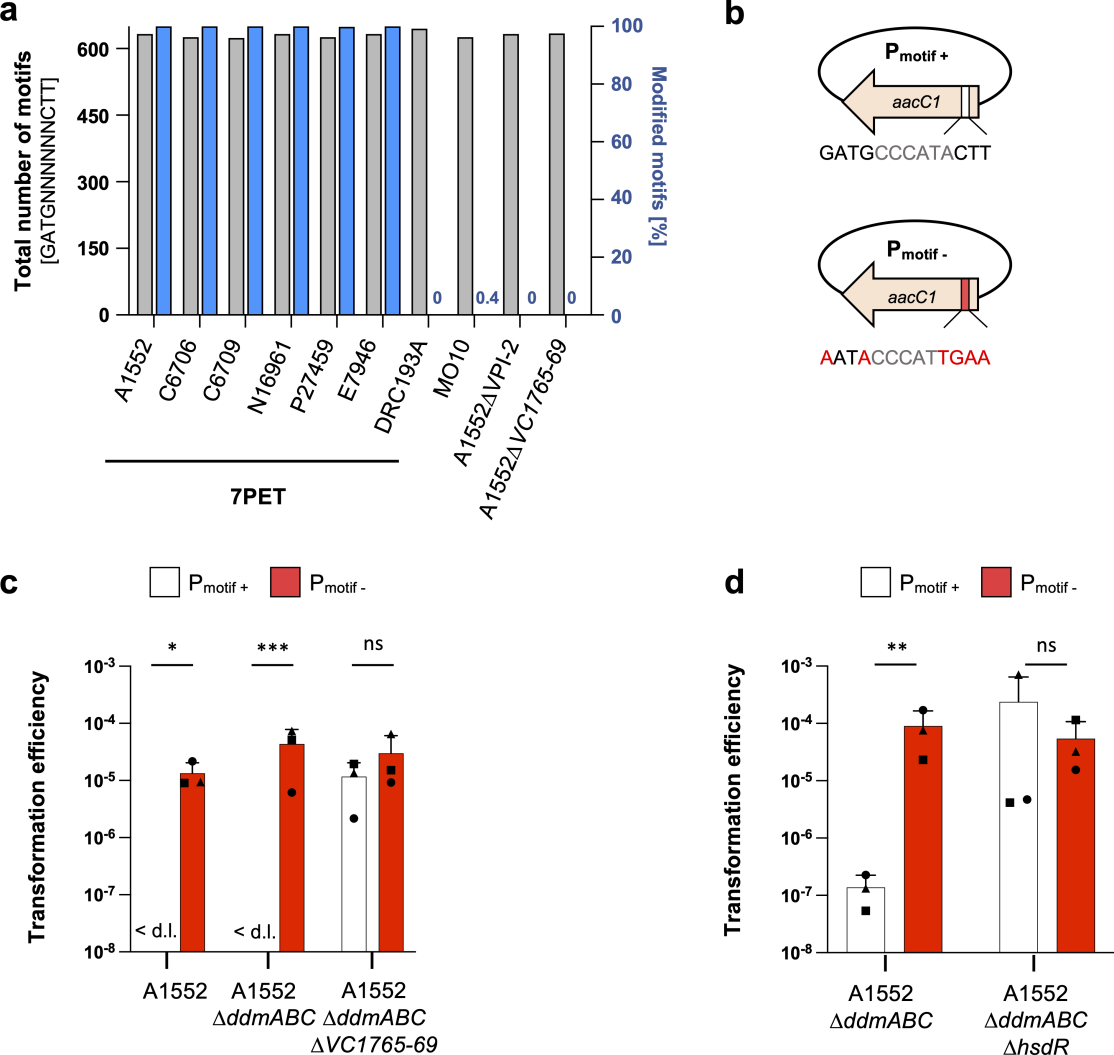
Type I RM system’s role in chromosomal methylation and plasmid restriction. (a) SMRT sequencing uncovers a distinctive modified DNA motif across various 7PET *V. cholerae* strains. The gray bars show the number of the DNA motif (GATGNNNNNNCTT) in each genome, while the blue bars denote the percentage of this motif methylated in each strain’s genome (see secondary y-axis on the right). (b) Diagrams of plasmid constructs. Two plasmids were engineered to either contain (P_motif+_) or lack (P_motif-_) the T1RM recognition motif. The latter plasmid was generated through the introduction of silent mutations. (c, d) The T1RM system hinders plasmid acquisition. Transformation assays compare the uptake of the two plasmids (P_motif+_ and P_motif-_) in strains A1552, A1552∆*ddmABC*, and A1552∆*ddmABC*∆*VC1765–69* (c) or A1552∆*ddmABC* and A1552∆*ddmABC*∆*hsdR* (d). Statistical differences were calculated on log-transformed data using a two-way analysis of variance corrected for multiple comparisons with Šidák’s method. *P < 0.05; **P < 0.01; ***P < 0.001; ns, not significant. <d.l., below detection limit.

If the T1RM system is active in *V. cholerae*, then we predicted that we should be able to detect a specific methylation signature that is absent in strains lacking this system. To test this hypothesis, we used SMRT PacBio whole-genome sequencing, which can detect the presence of various DNA modifications including methylation, to determine the methylomes of a selection of 7PET O1 serogroup strains (strains as in Fig. 1a), as well as those of control strains lacking the T1RM system (see Materials and Methods) (34–36). As shown in Fig. 2a, this analysis revealed a unique 13-nucleotide motif with methylation marks located on the second nucleotide within the sequence GATGNNNNNNCTT (m6A: GATGNNNNNNCTT:2). Upon further examination, we discovered that this DNA motif is present in over 600 copies throughout the genome of each strain and is modified in nearly 100% of cases in all O1 serogroup strains, except DRC193A (Fig. 2a). This phenotype is likely explained by the interruption of *hsdS* in this strain by an IS*256*-like transposase gene (37) (Fig. 1b). Finally, and as expected, the O139 serogroup strain MO10, which is missing the T1RM-encoding region of VPI-2 (Fig. 1a), and both a VPI-2 and a *VC1765–69*-deficient deletion strain (Table S1) all lacked this particular methylation mark (Fig. 2a).

### The T1RM impairs plasmid acquisition

Having identified the methylated recognition motif, we next tested the ability of this motif to target plasmids for restriction by the VPI-2 T1RM system. Serendipitously, we realized that the recognition sequence is present within the widely used gentamicin resistance cassette *aacC1*. We therefore created plasmid derivatives carrying *aacC1* either with the putative recognition sequence intact (P_motif+_) or with silent mutations that disrupt the nucleotide recognition sequence while preserving the protein coding sequence (P_motif-_) (Fig. 2b). We then purified these plasmids from *E. coli* and used them as substrates in an electroporation-based transformation assay to compare their transformation frequencies in various backgrounds. As shown in Fig. 2c, transformation with plasmid P_motif+_ was below the detection limit in the wild-type (WT) background (strain A1552) even though transformants could readily be obtained with plasmid P_motif-_. Furthermore, this disparity between the acquisition of the two plasmids became even stronger in the absence of the DdmABC system (23) (Fig. 2c), which is known to target derivatives of this high-copy number plasmid (38).

To determine if the plasmid restriction was mediated by the T1RM system, we removed either the entire five-gene restriction cluster or only the *hsdR* gene from the A1552∆*ddmABC* background and then assessed the plasmid transformability of the resulting strains. As shown in Fig. 2c and d, both deletions led to the recovery of P_motif+_ transformants. Moreover, the transformation difference between the P_motif+_ and P_motif-_ plasmids was now no longer statistically significant. Consequently, we conclude that the T1RM system is active, that it methylates a specific recognition sequence, and that when this sequence is present on non-self DNA, the acquisition of this non-methylated DNA is restricted in an HsdR-dependent manner.

### Genes embedded in the T1RM cluster protect against phages with modified genomes

**Fig 3:**
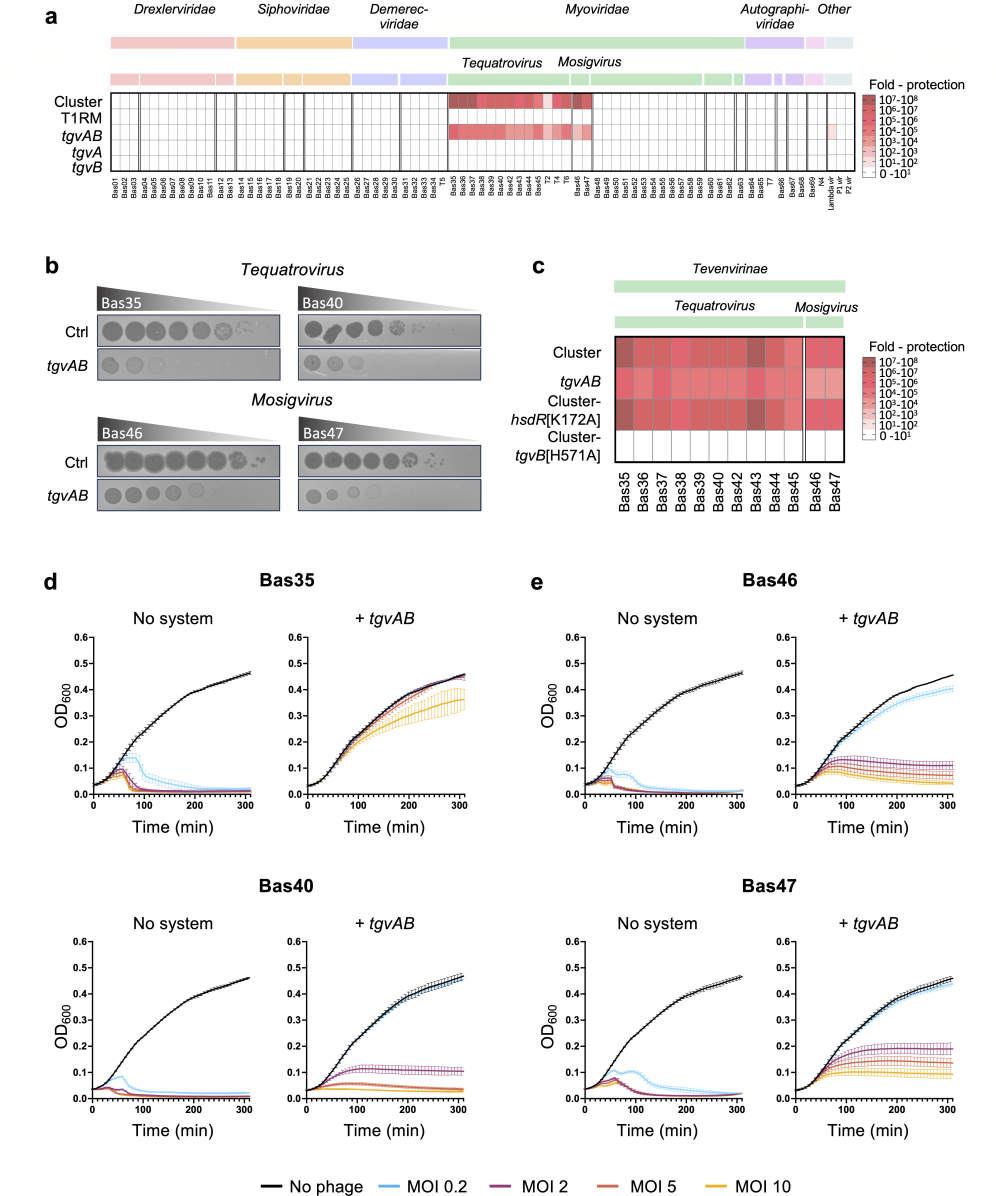
Protection against *Tevenvirinae* by Tgv proteins encoded by the T1RM-embedded genes. (a) Observed defense activity against the BASEL phage collection. Protection levels (fold protection, as shown by the color code on the right) were determined by comparing plaque formation in strains with the system to those without, using 10-fold serial dilution assays. Data represent the average of two replicates. (b) Phage plaque assays on *E. coli* strains harboring an empty transposon (control, Ctrl) or the two T1RM-embedded genes (*tgvAB*), using a 10-fold serial dilution. (c) Defense against *Tevenvirinae* does not depend on HsdR’s restriction activity. Protection against members of the *Tevenvirinae* was assessed in *E. coli* strains expressing either the native five-gene cluster or only *tgvAB*, as shown in panel a. Additionally, strains containing the five-gene cluster with site-directed mutations *hsdR*[K172A] and *tgvB*[H571A] were evaluated to ascertain the individual contributions of the T1RM and TgvAB systems to defense capability. Data represent the average of three replicates. Details as in panel a. (d, e) Growth curves of *E. coli* cultures carrying an empty transposon (no system) or Tn*tgvAB* (+ *tgvAB*), without (NO phage) or with exposure to phages, initiated at time 0 with various multiplicities of infection (MOIs) (0.2, 2, 5, or 10). (d) *Tequatroviruses* and (e) *Mosigviruses* were used for infection. The presented data are the average of three independent experiments (±SD, illustrated with error bars).

Type I restriction-modification systems are recognized for their important role in defending the cell against phage infection (39). Therefore, we aimed to investigate the ability of the entire RM cluster, including the two embedded genes, to protect against viral infections. However, given that commonly used *Vibrio* phages, such as ICP1, ICP2, and ICP3, are typically isolated using VPI-2-carrying 7PET strains as the host [for example, strain E7946 and its derivatives (40)] and that they coevolved with these strains in cholera endemic areas (41, 42), it is unlikely that any defense system encoded on VPI-2 would provide protection against these phages, potentially due to the presence of phage-encoded anti-defense mechanism. Therefore, we engineered the *E. coli* strain MG1655 to carry an arabinose-inducible version of the entire five-gene RM cluster (*VC1769–65*), which was integrated into its chromosome. Utilizing this strain and a strain without the cluster as a control, we screened for protection against the BASEL collection, a recently established phage collection that represents the natural diversity of *E. coli* phages (43). As shown in Fig. 3, we noted a reduction in the efficiency of plaquing of at least 1,000-fold compared to the non-defense control upon infection with members of the *Tevenvirinae* subfamily. The *Tevenvirinae* subfamily is characterized by their unique cytosine modifications, which play a crucial role in their defense against RM systems like the T1RM (43). Specifically, *Tequatrovirus* group phages feature cytosines that are hydroxymethyl-glucosylated, while *Mosigviruses* possess cytosines that are hydroxymethyl-arabinosylated (10, 44).

To determine which part of the RM cluster was responsible for this protection, we created *E. coli* strains that either carried the T1RM cluster or just the embedded two-gene cluster independently. Strikingly, this revealed that the two-gene cluster was responsible for this protection (Fig. 3a and b). Furthermore, the two genes did not provide protection when expressed individually, indicating a necessity for their combined action to achieve the observed anti-phage activity (Fig. 3a). For reasons explained below, we named these two genes as type I-embedded GmrSD-like system of VPI-2, *tgvA* (*VC1767*) and *tgvB* (*VC1766*).

Interestingly, the strain carrying only the *tgvAB* genes showed significantly less protection (between 10- and 100-fold reduction) against several tested phages compared to the strain harboring the entire gene cluster (Fig. 3a and c). To investigate if restriction by the T1RM system contributes to this increased protection, we engineered a variant of the five-gene cluster containing a site-directed mutant of *hsdR* designed to inactivate restriction. Specifically, we replaced the catalytic lysine in the PD-(D/E)XK nuclease motif of the encoded HsdR protein with alanine (HsdR[K172A]), a modification known to abolish restriction activity in other T1RM systems (45–49). Notably, this mutated construct displayed a similar protection pattern to the unmodified gene cluster (Fig. 3c), indicating that the T1RM’s restriction activity does not play a direct role in the anti-phage defense. A plausible explanation for the reduced effectiveness of the standalone *tgvAB* operon, compared to its performance within the entire gene cluster, could be an imbalance in the stoichiometry of TgvA and TgvB proteins. This imbalance might be caused by the use of the artificial *P*_BAD_ promoter and a non-native ribosome-binding site upstream of *tgvA* in the two-gene operon only construct.

The reasons for the lack of protection against the BASEL phages by the T1RM system, despite the presence of the recognition motif in 95.6% of these phages (Table S2), might be attributable to multiple factors. RM systems are the most prevalent defense mechanisms in bacterial genomes (5). As a result, many phages have evolved counter-defense mechanisms. For instance, phage T7 produces an Ocr (overcomes classical restriction) protein that mimics DNA to sequester RM enzymes, thereby preventing the restriction of its own DNA (50–52). Furthermore, the initial characterization of the BASEL phage collection by Maffei *et al*. showed that the T1RM systems tested were largely ineffective against phages outside the *Drexlerviridae* family (43). Closer inspection of their findings showed that fewer than 10% of the phages in the BASEL collection were significantly restricted (i.e., by a factor of 10 or more) by the tested T1RM systems, despite approximately 80% of the phages’ genomes containing the cognate recognition sites. Thus, our findings align with these previous observations.

To dissect the underlying mechanism of anti-phage defense by TgvAB, we monitored the growth kinetics of *E. coli* strains infected with increasing multiplicities of infection (MOIs) for both *Tequatrovirus* (Fig. 3d) and *Mosigvirus* phages (Fig. 3e). As expected, cultures of the no system control strain grew and then lysed in an MOI-dependent manner (Fig. 3d and e). In contrast, TgvAB producing cultures infected with the *Tequatrovirus* Bas35 continued to grow at rates indistinguishable from those of the no phage control up to and including MOI 5, before being partially overcome at MOI 10 (Fig. 3d). This phenotype is consistent with TgvAB acting directly to target the invading phage. However, TgvAB producing cultures infected with either the *Tequatrovirus* Bas40 or the *Mosigviruses* Bas46 and 47 all showed more variable levels of protection (Fig. 3d and e). Indeed, while protection was robust at MOI 0.2, at higher MOIs, we observed growth inhibition and even partial lysis. Nevertheless, given that the cultures mostly continued to grow past the point at which they lysed in the no system control, together with the direct protection observed against Bas35 at all tested MOIs, we conclude that TgvAB likely also acts directly against these phages, but that they are better able to overwhelm the system at high MOI.

### The TgvAB defense system is a member of the GmrSD family of type IV restriction enzymes

**Fig 4:**
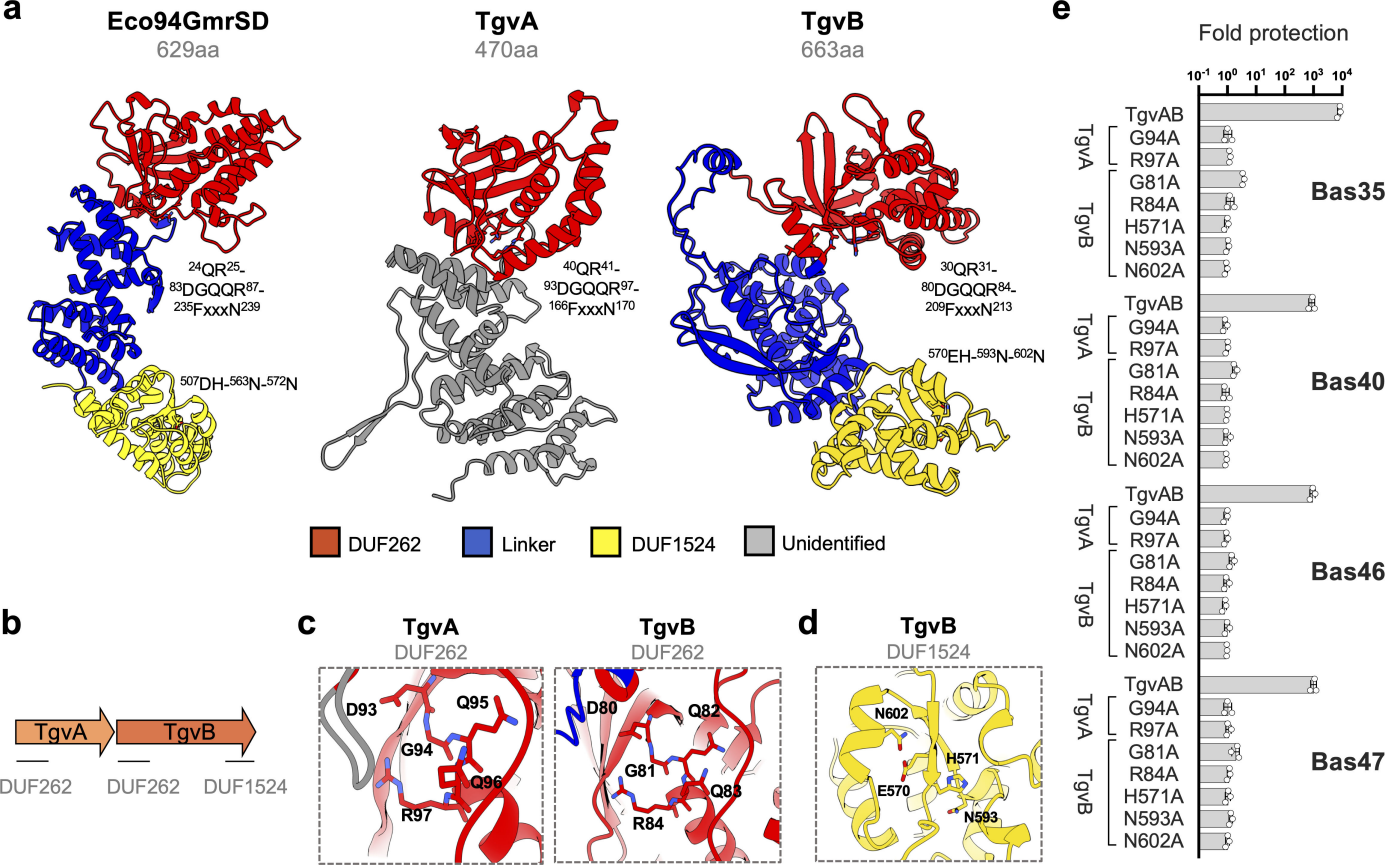
The two-protein TgvAB defense system is a member of the GmrSD family of type IV restriction enzymes. (a) Structural models of Eco94GmrSD of *E. coli* STEC_94C and TgvA (*VC1767*) and TgvB (*VC1766*) of *V. cholerae* 7PET strains. The models, produced via AlphaFold (ColabFold), portray the domains with corresponding colors, while also highlighting the residues characteristic to the DUF262 and DUF1524 domains. Images were generated using ChimeraX 1.7.1. (b) Schematics displaying conserved domains identified in the TgvA and TgvB proteins. (c, d) Zoomed view of the conserved (c) DGQQR motif found in the DUF262 region of TgvA and TgvB and (d) of the His-Me finger motif within the DUF1524 of TgvB, highlighting the catalytic histidine (H) situated at the terminus of the β1 strand, the Asparagine (N) residue positioned in the loop region and the final N residue within the α-helix. (e) Site-directed mutagenesis removed the antiviral effect. The level of protection was evaluated as described in Fig. 3. Mutagenesis aimed at disrupting NTPase or endonuclease functions exerted by DUF262 and DUF1524, respectively. The data are averages from three independent experiments (±SD, as shown by the error bars).

Bioinformatic analysis of the TgvAB system revealed that TgvA (*VC1767*) and TgvB (*VC1766*) both possess an N-terminal DUF262 domain, while TgvB additionally contains a C-terminal DUF1524 domain (Fig. 4a and b). Interestingly, previous work by Machnicka *et al.* found that GmrS and GmrD proteins contain the DUF262 and DUF1524 domains, respectively, typically coming together to form GmrSD fusion proteins (53). Notably, the TgvB homolog from classical biotype *V. cholerae* (VC0395_A1364) was also identified as a GmrSD homolog in this study (53). These double domain forms of GmrSD function as modification-dependent type IV restriction enzymes, and are known to specifically recognize and cleave DNA containing sugar-modified hydroxymethyl cytosines. However, they exhibit no activity against unmodified DNA (53–56). Given that such modifications are typical of the *Tevenvirinae* (10) and the specific protective effect we observed against them (Fig. 3), this suggests that TgvAB may function in a similar manner. Importantly, and in contrast to classical single protein GmrSD such as Eco94GmrSD (Fig. 4a) (54), our phage infection assay revealed that TgvA and TgvB cannot function independently, and that both proteins are required for anti-phage activity.

Machnicka *et al.* showed that the predominant form of GmrSD is as a single multi-domain protein containing an N-terminal DUF262(GmrS) domain and a C-terminal DUF1524(GmrD) domain, separated by an alpha helical linker region (53). This domain organization was subsequently confirmed by crystal structures of the related GmrSD family members BrxU, which also recognizes and degrades DNA containing modified cytosines, and the phosphorothioate modification sensing enzyme SspE (57–59). Furthermore, biochemical experiments with these enzymes have shown that the N-terminal DUF262 likely functions as DNA modification sensor, and uses nucleotide binding and hydrolysis to regulate the activity of the C-terminal DUF1524, which functions as a nuclease to degrade non-self DNA (57, 59). Strikingly, structural modeling of Eco94GmrSD and TgvAB using AlphaFold (60) revealed that TgvB is predicted to share a similar domain architecture, although in the case of TgvA, this similarity is limited to the N-terminal DUF262 domain (Fig. 4a and b). Moreover, the top hits in structural alignments of the TgvAB models were SspE and BrxU, reinforcing the idea that these proteins are related.

Next, to further investigate the relative contributions of the DUF262 and DUF1524 domains to TgvAB function, we used the structural modeling and alignments to identify key residues in each domain. For both TgvA and B, the three highly conserved motifs characteristically associated with the DUF262 domain [i.e., (i) QR, (ii) DGQQR, and (iii) FxxxN] were readily identifiable (Fig. 4a through d) (53). Notably, the DGQQR motif is thought to form part of a nucleotide-binding pocket and to be required for nucleotide hydrolysis. Indeed, site-directed mutants of either TgvA or TgvB encoding substitutions in this motif previously shown to disrupt NTPase activity (57–59) all resulted in a total loss of anti-phage activity (Fig. 4c through e). In contrast, the DUF1524 domain contains a highly conserved H…N…H/N motif, which belongs to the His-Me finger nuclease superfamily and that assumes a characteristic ββα fold (61, 62). Such a motif was readily apparent in the C-terminal domain of the predicted TgvB structure, and consistent with previous findings (54, 57–59), substitutions designed to disrupt either the catalytic histidine (TgvB[H571A]) or the metal-binding asparagine (TgvB[N602A]) were sufficient to abolish anti-phage activity (Fig. 3c, 4d and e).

Overall, our results suggest that the TgvAB system senses phages with hypermodified cytosines in a manner that requires the DUF262 domains of both TgvA and B, and that the His-Me nuclease domain of TgvB likely functions as the effector against phage DNA. The notion that TgvAB recognizes modified DNA aligns with findings from Gomez and Waters, who concurrently described the TgvAB system in their study (63). They demonstrated that T2/T4 phages lacking functional glucosyltransferase genes, essential for the glucosylation of hydroxymethylated cytosines, could evade the defense exerted by TgvAB (63). This further supports the specificity of TgvAB toward modified DNA. Nevertheless, why TgvB alone is not sufficient for phage protection remains unclear. One possibility is that TgvA is required to overcome a phage encoded inhibitor. For example, some GmrSD family enzymes such as Eco94GmrSD are inhibited by the protein IPI*, which is co-injected into the host cell with the T4 genome (54, 55, 64). However, TgvA could equally also play a regulatory or structural role, and further work will therefore be needed to clarify these possibilities.

### Occurrence of the *tgvAB* system within and outside T1RM clusters

**Fig 5:**
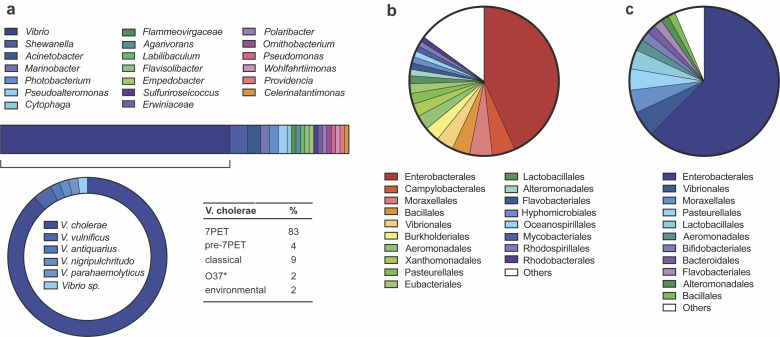
Phylogenetic distribution of the restriction systems. (a) The presence of the five-gene cluster (*VC1765–69*) was assessed across 41,142 bacterial genomes. The results revealed its distribution beyond *V. cholerae*, which represented 65.8% of all hits. *V. cholerae O37 serogroup strains are known to be closely related to classical O1 strains with highly similar chromosomal backbones. (b, c) Exploration of the (b) T1RM system (*VC1769-68-65*) and (c) TgvAB system (*VC1767–66*) across the bacterial genomes demonstrates their assessment at the order level of taxonomy. Orders represented in less than 1% of instances were consolidated into a singular category labeled “Others” for the visualization. For details at the species level, see Tables S3 to S5.

To investigate the prevalence of *tgvAB* homologs within the T1RM cluster, we examined the distribution of the specific five-gene operon within 41,142 bacterial genomes (see Materials and Methods for details). This *in silico* analysis revealed that the gene architecture found in VPI-2 of *V. cholerae* is also present in a variety of other bacterial genera (Fig. 5a) with 79 hits within this genome database, including *Shewanella, Acinetobacter*, and *Pseudoalteromonas* species (see Table S3 for species-level details). This wider distribution indicates the potential functional conservation of these gene arrangements across different gram-negative bacteria. However, the genus *Vibrio* was still most prominently featured in these findings with 52 hits (Fig. 5a). Precisely, apart from *V. cholerae*, species such as *Vibrio vulnificus*, *Vibrio antiquarius, Vibrio nigripulchritudo, Vibrio parahaemolyticus,* and the unclassified *Vibrio* strain B1ASS3 (*Vibrio* sp.) were identified to carry similar gene clusters (Fig. 5a). Despite the presence of these diverse *Vibrio* species, *V. cholerae* 7PET strains were the most commonly identified with 38 hits (Fig. 5a), likely reflecting their prominent representation in the NCBI database.

Subsequent analysis focused on the independent occurrences of the T1RM and TgvAB systems. As expected, the T1RM system was widespread (2,808 hits) across numerous bacterial orders (Fig. 5b and Table S4 for species-level details). Homologs of the *tgvAB* operon alone were slightly less common with 1,341 hits (Table S5 for species-level details), yet 17 times more prevalent than the instances of the five-gene operon described above. Indeed, as shown in Fig. 5c, the occurrence of TgvAB homologs spans a wide array of bacterial orders, with species found in the human gut, like *Bacteroides fragilis* (Bacteroidales), to organisms isolated from permafrost, such as *Psychrobacter cryohalolentis* (Moraxellales).

### Conclusion

In this study, we aimed to characterize the predicted restriction gene cluster of VPI-2. We showed that the T1RM system actively methylates the genomes of 7PET *V. cholerae* strains, while restricting unmethylated foreign DNA. Additionally, we identified a novel two-protein modification-dependent restriction system, TgvAB, which is embedded within the T1RM cluster. Interestingly, Picton *et al.* demonstrated that the TgvB homolog BrxU, along with the bacteriophage exclusion (BREX) system (65), works in concert to offer complementary resistance against both modified and non-modified phages (53, 57). Therefore, it is tempting to speculate that the embedding of the *tgvAB* operon within the T1RM cluster serves a similar complementary role in *V. cholerae*. Supporting this notion, Machnicka *et al.* noted that GmrSD homologs are frequently encoded within type I RM loci. An example includes the gene encoding the DUF262 domain-containing protein RloF of *Campylobacter jejuni*, which is situated between *hsdR* and *hsdS* of a T1RM operon (66), similar to the positioning of *tgvAB* described in this study. That defense systems tend to cluster together within defense islands has been established over several years (11, 12, 67). However, this concept was recently extended by Payne and colleagues by identifying specific genes embedded within multi-gene defense clusters, highlighting the complex organization and integration of these systems within bacterial genomes (68). Notably, their research found GmrSD-like genes embedded within Hma (helicase, methylase, ATPase) defense gene clusters. However, unlike the HEC-05 (= BrxU) and HEC-06 GmrSD-like proteins identified in their work, which function independently (57, 68), our findings indicate that the TgvAB defense operates as a two-protein system, underscoring the diversity in bacterial defense strategies.

## MATERIALS AND METHODS

### Bacterial strains, plasmids, and culture conditions

The bacterial strains and the plasmids used in this study are listed in Table S1. pUC18-mini-Tn7T-Gm-*lacZ* was a gift from Herbert Schweizer via Addgene plasmid #63120 (69). The primary *V. cholerae* strain used, A1552, is a fully sequenced toxigenic O1 El Tor Inaba strain, representing the ongoing seventh cholera pandemic (70, 71). Unless stated otherwise, bacteria were aerobically cultured in lysogeny broth (LB; 1% tryptone, 0.5% yeast extract, 1% sodium chloride; Carl Roth, Switzerland) with shaking at 180 rpm, or on LB agar plates at either 30°C or 37°C. When required, antibiotic selection was applied using ampicillin (100 µg/mL), kanamycin (75 µg/mL), and gentamicin (25 or 50 µg/mL). For natural transformation, chitin powder (Alfa Aesar via Thermo Fisher, USA) was combined with half-concentrated Instant Ocean medium (Aquarium Systems) and sterilized by autoclaving prior to adding the bacterial cultures.

Conjugation with MFDpir (72) was used to introduce the mini-Tn7 transposon derivatives into *E. coli* strain MG1655 on agar plates containing 0.3 mM diaminopimelic acid (Sigma-Aldrich). To induce expression from the *P*_BAD_ promoter, cultures were grown in media containing 0.2% L-arabinose. For bacteriophages experiments, LB medium was supplemented with 5 mM CaCl_2_ + 20 mM MgSO_4_. Double-layer LB plates were prepared by adding 0.5% agar for semi-solid agar and 1.5% agar for the solid base.

### Genetic engineering of strains and plasmids

Standard molecular cloning techniques were utilized for the cloning process (73) using the following enzymes: Pwo polymerase (Roche), Q5 High-Fidelity Polymerase (New England Biolabs), GoTaq Polymerase (Promega), restriction enzymes (New England Biolabs), and T4 DNA ligase (New England Biolabs). Enzymes were used according to the manufacturer’s instructions. All constructs were verified through PCR and/or Sanger or Nanopore sequencing (performed by Microsynth AG, Switzerland) and analyzed using SnapGene version 4.3.11.

*V. cholerae* strains were created through natural transformation and Flp recombination (TransFLP) (74–76) or through allelic exchange using derivatives of the suicide plasmid pGP704-Sac28 (77) and SacB-based counter-selection on NaCl-free LB plates with 10% sucrose. Mini-Tn7 transposons, containing *araC* and the gene(s) of interest regulated by the arabinose-inducible promoter *P*_BAD_, were inserted in *E. coli* downstream of *glmS* via triparental mating, following established protocols (78). Site-directed mutations in these constructs were introduced by inverse PCR prior to their transposition into the *E. coli* chromosome.

### PacBio (SMRT) sequencing

Genomic DNA was purified from overnight cultures using Qiagen’s Genomic-tip procedure combined with the Genomic DNA buffer set (Qiagen, Switzerland), following the manufacturer’s instructions. Sample processing, PacBio Single Molecule, Real-Time (SMRT) sequencing, and *de novo* genome assembly were performed at the University of Lausanne’s Genomic Technology Facility, as previously described (34). Note that the assembled genomes of strains A1552, C6706, C6709, P27459, E7946, DRC193A, and MO10 have been previously reported without analysis of their epigenetic modifications (34, 35, 71).

### Electroporation-mediated transformation of *V. cholerae* using plasmids

To explore the T1RM system’s efficiency in restricting DNA with specific recognition sequences, we compared the uptake frequency of a plasmid harboring the putative recognition motif (P_motif+_) to that of a variant plasmid with silent mutations in *aacC1* (P_motif-_) altering its sequence while maintaining the encoded aminoglycoside-3-*O*-acetyltransferase-I protein. Transformation frequencies were assessed through electroporation. *V. cholerae* competent cells were prepared by standard protocols (73), involving 1:100 dilution of overnight cultures, growth for 2 h and 30 min at 37°C (until an optical density at 600 nm [OD_600_] of ~1.0), and washing steps with cold 2 mM CaCl_2_ and 10% glycerol before shock-freezing. After 2 h at −80°C, electroporation with 300 ng plasmid was performed at 1.6 kV followed by recovery in 2xYT-rich medium at 30°C for 2  h. Cells were plated on LB agar with and without kanamycin and incubated at 37°C overnight. Transformation frequencies were calculated as the ratio of kanamycin-resistant transformants to the total number of bacteria.

### Bacteriophage handling and culturing

The *E. coli* BASEL phage collection (43) was used in this study. To generate phage stocks, an *E. coli* MG1655Δ*araCBAD* (79) overnight culture was diluted and grown to the exponential phase in LB medium supplemented with 5 mM CaCl_2_ and 20 mM MgSO_4_. Subsequently, the culture was 1:10 diluted in prewarmed medium, infected with 10^4^ plaque-forming units/mL, and incubated under shaking conditions at 37°C for 5 h. Following incubation, centrifugation and filtration were used to clear the lysate, which was then treated with 1% chloroform and stored at +4°C. Phage titers were determined using plaque assays on the propagation strain.

### Bacteriophage plaque assays

For plaque assays, *E. coli* MG1655Δ*araCBAD*, either with the candidate defense system or the empty mini-Tn7 transposon control, was grown in LB medium. Overnight cultures were diluted 1:100 in LB medium supplemented with 0.2% arabinose, 5 mM CaCl_2_, and 20 mM MgSO_4_ and grown at 37°C with shaking for 2 h. Once reaching the exponential phase, the cultures were diluted 1:40 in 0.5% LB agar containing 5 mM CaCl_2_, 20 mM MgSO_4_, and 0.2% arabinose, then overlaid on 1.5% LB agar. Phage samples were serial diluted in LB medium with 5 mM CaCl_2_ and 20 mM MgSO_4_ and spotted onto the bacterial overlays. After overnight incubation at 37°C, plaques were counted to assess the defense system’s effectiveness compared to the mini-Tn7-carrying control strain (= fold protection).

### Infection kinetics

The infection kinetics assay of *Tequatroviruses* (Bas35, Bas40) and *Mosigviruses* (Bas46, Bas47) was conducted as follows: overnight cultures of *E. coli* strains were diluted 1:100 in LB medium supplemented with 5 mM CaCl_2_, 20 mM MgSO_4_, and 0.2% arabinose. Bacterial cultures were then incubated at 37°C with shaking for 2 h. Subsequently, 20 µL of phage per well at MOIs of 0, 0.2, 5, or 10 was added in technical triplicate to a 96-well plate. The cultures were further diluted 1:10 in the same LB condition, and 180 µL of each diluted culture was then added to the wells. The SpectraMax i3x plate reader from Molecular Devices was utilized to assess bacterial growth at 37°C, with measurements taken at 6-minute intervals over a total of 49 cycles. To calculate the MOI, cultures of strains MG1655∆*araCBAD*-TnAraC (no system control) and MG1655∆*araCBAD*-TnTgvAB were cultured following the protocol outlined in the Bacteriophage Plaque Assays section. Colony-forming units (per milliliter) were quantified by spotting serially diluted cultures onto LB plates. The calculated values represent the average of three technical replicates.

### Bioinformatics analyses

The VPI-2 genomic region of 7PET O1 strains and one O139 serogroup strain (MO10) was compared and visualized using Clinker software (v.0.0.25, default parameters) (80) after reannotation of the genome sequence of strain A1552 using the Prokaryotic Genome Annotation pipeline version 2023-10-03.build7061 (81) to unify the annotation method. For sequence similarity, NCBI’s blastp was utilized (default parameters, non-redundant protein database; accession August 2023), while structural modeling was conducted with ColabFold (1.5.2) (82) based on AlphaFold2 (60) using default settings. The DALI server was employed for structural similarity predictions against the Protein Data Bank (83, 84).

The distribution of the specific five-gene operon (*VC1769–6*5) across bacterial species was examined with MacSyFinder (v.2.1) (85), using a comprehensive database of sequenced and fully assembled bacterial genomes (taxid:2) from the NCBI database (accession date 26 January 2024; GenBank database with all complete and chromosome level assemblies using the data sets utility from the NCBI command line tool). This analysis therefore covered a data set comprising 41,142 bacterial genomes, which altogether contained over 154 million protein sequences.

To build hidden Markov model (HMM) profiles for each target coding sequence within the *VC1769–6*5 operon, homologous protein sequences were identified via PSI-BLAST searches in the NCBI database (three iterations), using the non-redundant protein sequence database (accessed in February 2024) with a cutoff e-value of 1e − 10.

After identifying homologous sequences for each CDS through PSI-BLAST, the sequences were aligned using MAFFT (v.7.508, --maxiterate 1000 –localpair parameters for higher accuracy alignments) (86). From these multiple alignments, HMM profiles were generated with HMMER (v.3.3.2, using hmmbuild with default parameters) (87), forming the basis for constructing different models in MacSyFinder. These models were used to search for the occurrence of the CDS in various combinations encompassing the T1RM and/or TgvAB system genes. The constructed models were then applied in a search across the bacterial protein database mentioned above.

### Statistics and reproducibility

Results are derived from biologically independent experiments, as specified in the figure legends. Statistical analyses were conducted using Prism software (v.10.2.1; GraphPad).

## Data Availability

All SMRT sequencing raw data have been made available on Zenodo (three data sets: 10.5281/zenodo.10838595; 10.5281/zenodo.10839511; 10.5281/zenodo.10839547).

## SUPPLEMENTAL MATERIAL

Table S1 jb.00145-24-s0001.docx Bacterial strains and plasmids used in this study.

Table S2 jb.00145-24-s0002.xlsx Counts of the T1RM recognition motif in phage genomes from the BASEL collection.

Table S3 jb.00145-24-s0003.xlsx Summary of matching hits for the T1RM + TgvAB (VC1769-65) model, detected by MacSyFinder v.2.

Table S4 jb.00145-24-s0004.xlsx Summary of matching hits for the T1RM (VC1769-68-65) model, detected by MacSyFinder v.2.

Table S5 jb.00145-24-s0005.xlsx Summary of matching hits for the TgvAB (VC1767-66) model, detected by MacSyFinder v.2.

ASM does not own the copyrights to Supplemental Material that may be linked to, or accessed through, an article. The authors have granted ASM a non-exclusive, world-wide license to publish the Supplemental Material files. Please contact the corresponding author directly for reuse.
