# Supplementary material for: Vibrio cholerae pathogenicity island 2 encodes two distinct types of restriction systems
Source: J Bacteriol. 2024 Aug 12;206(9):e00145-24. doi: 10.1128/jb.00145-24 (PMC11411939; doi:10.1128/jb.00145-24)
Supplement: Table S1 — Bacterial strains and plasmids used in this study. [file jb.00145-24-s0001.docx]

**Supplementary Table 1. Bacterial strains and plasmids used in this study.**

| **Strains or plasmids** | **Genotype/description^a, b^** | **Strain number** | **Reference** |
| --- | --- | --- | --- |
| ***Vibrio cholerae*** | | | |
| A1552 | Wild type; O1 El Tor Inaba; isolated in 1992, Peruvian origin; Rif^R^ | MB#1 | (1, 2) |
| C6706 | O1 El Tor Inaba; isolated in 1991, Peru; original isolate; non-mutated *luxO*); kind gift from J.J. Mekalanos | MB#4522 | (3) |
| C6709 | O1 El Tor Inaba; isolated in 1991, Peru; Strep^R^ | MB#1503 | (4) |
| N16961 | O1 El Tor Inaba; isolated in 1975, Bangladesh; *hapR* frame-shifted; Strep^R^ | MB#2 | (1) |
| P27459 | O1 El Tor Inaba; isolated in 1976, Bangladesh; Strep^R^ | MB#1504 | (5) |
| E7946 | O1 El Tor Ogawa; isolated in 1978, Bahrain; Strep^R^ | MB#2600 | (6) |
| DRC193A | O1 El Tor; isolated in 2011, Democratic Republic of Congo; Strep^R^ | MB#1954 | (7) |
| MO10 | O139 strain isolated in 1992, India; *hapR* mutated (HapR[R12L]); Strep^R^ | MB#5 | (8) |
| A1552∆VPI-2 | A1552 deleted for entire VPI-2 through TransFLP; A1552∆VPI-2::FRT; Rif^R^ | MB#10090 | This study |
| A1552-VPI-2∆#2 (∆*VC1765-1769*) | A1552 with deleted region #2 of VPI-2 (∆*VC1765-1769*::FRT-*kan*-FRT); Kan^R^, Rif^R^ | MB#8678 | (9) |
| A1552∆*ddmABC* | A1552Δ*ddmABC*-noFRT; *ddmABC* deleted using suicide plasmid pGP704∆*ddmABC*; Rif^R^ | MB#9747 | This study |
| A1552∆*ddm*ABC  ∆*VC1765-1769*::  FRT-*kan*-FRT | A1552∆*ddmABC* with deleted *VC1765-1769;* ∆*VC1765-1769*::FRT-*kan*-FRT; Kan^R^, Rif^R^ | MB#11377 | This study |
| A1552∆*ddmABC*  ∆*VC1765-1769* | A1552∆*ddmABC*∆*VC1765-1769*::FRT-*kan*-FRT after flip and cure resulting in A1552∆*ddmABC*∆*VC1765-1769*::FRT; Rif^R^ | MB#11379 | This study |
| A1552∆*ddmABC*  ∆*hsdR*::FRT-Kan-FRT | A1552∆*ddmABC* deleted for *VC1765* (*hsdR*); ∆*VC1765*::FRT-*kan*-FRT; Kan^R^, Rif^R^ | MB#11897 | This study |
| A1552∆*ddmABC*  ∆*hsdR* | A1552∆*ddmABC*∆*hsdR*::FRT-*kan*-FRT after flip and cure resulting in A1552∆*ddmABC* ∆*VC1765*::FRT; Rif^R^ | MB#11898 | This study |
| ***Escherichia coli*** | | | |
| S17-1λ*pir* | TpR SmR *thi pro hsdR*- *hsdM*+ *recA* RP4-2-Tc::Mu-Km::Tn7 (λ*pir*) | MB#648 | (10) |
| MFD*pir* | MG1655 RP4-2-Tc::[∆Mu1::*aac(3)IV*-∆*aphA*- ∆*nic*35-∆Mu2::*zeo*] ∆*dapA*::(*erm*-*pir*) ∆*recA* | MB#4662 | (11) |
| TOP10 | F- *mcrA* ∆(*mrr-hsdRMS*-*mcrBC*) Φ80*lacZ*∆M15 ∆*lac*X74 *recA1 araD*139 ∆(*ara leu*) 7697 *galU galK rpsL* (Strep^R^) *endA*1 *nupG* | MB#741 | Invitrogen |
| MFD*pir* / pGP704-TnAraC | MFD*pir* carrying pGP704-Tn with *araC* and *P*_BAD_; Amp^R^, Gent^R^ | MB#8793 | This study |
| MFD*pir* / pGP704-Tncluster | MFD*pir* carrying pGP704-TnCluster; Amp^R^, Gent^R^ | MB#11371 | This study |
| MFD*pir* / pGP704-TnT1RM | MFD*pir* carrying pGP704-TnT1RM; Amp^R^, Gent^R^ | MB#11367 | This study |
| MFD*pir* / pGP704-Tn*tgvAB* | MFD*pir* carrying pGP704-Tn*tgvAB*; Amp^R^, Gent^R^ | MB#11368 | This study |
| MFD*pir* / pGP704-Tn*tgvA* | MFD*pir* carrying pGP704-Tn*tgvA*; Amp^R^, Gent^R^ | MB#11369 | This study |
| MFD*pir* / pGP704-Tn*tgvB* | MFD*pir* carrying pGP704-Tn*tgvB*; Amp^R^, Gent^R^ | MB#11370 | This study |
| MG1655∆*araCBAD* | F- λ-, rph-1 ∆*araCBAD* | MB#4441 | (12) |
| MG1655∆*araCBAD*-Tnempty | MG1655∆*araCBAD* with integrated Tn*araC*; Gent^R^ | MB#10858 | This study |
| MG1655∆*araCBAD*-TnT1RM | MG1655∆*araCBAD* with integrated TnT1RM; Gent^R^ | MB#11372 | This study |
| MG1655∆*araCBAD*-TnCluster | MG1655∆*araCBAD* with integrated TnCluster; Gent^R^ | MB#11373 | This study |
| MG1655∆*araCBAD*-Tn*tgvAB* | MG1655∆*araCBAD* with integrated Tn*tgvAB*; Gent^R^ | MB#11374 | This study |
| MG1655∆*araCBAD*-Tn*tgvA* | MG1655∆*araCBAD* with integrated Tn*tgvA*; Gent^R^ | MB#11375 | This study |
| MG1655∆*araCBAD*-Tn*tgvB* | MG1655∆*araCBAD* with integrated Tn*tgvB*; Gent^R^ | MB#11376 | This study |
| MFD*pir* / pGP704-Tn*tgvA*[G94A]*B* | MFD*pir* carrying pGP704-Tn*tgvA*[G94A]*B*; Amp^R^, Gent^R^ | MB#11382 | This study |
| MFD*pir* / pGP704-Tn*tgvA*[R97A]*B* | MFD*pir* carrying pGP704-Tn*tgvA*[R97A]*B*; Amp^R^, Gent^R^ | MB#11383 | This study |
| MFD*pir* / pGP704-Tn*tgvAB*[G81A] | MFD*pir* carrying pGP704-Tn*tgvAB*[G81A]; Amp^R^, Gent^R^ | MB#11380 | This study |
| MFD*pir* / pGP704-Tn*tgvAB*[R84A] | MFD*pir* carrying pGP704-Tn*tgvAB*[R84A]; Amp^R^, Gent^R^ | MB#11381 | This study |
| MFD*pir* / pGP704-Tn*tgvAB*[H571A] | MFD*pir* carrying pGP704-Tn*tgvAB*[H571A]; Amp^R^, Gent^R^ | MB#11384 | This study |
| MFD*pir* / pGP704-Tn*tgvAB*[N593A] | MFD*pir* carrying pGP704-Tn*tgvAB*[N593A]; Amp^R^, Gent^R^ | MB#11385 | This study |
| MFD*pir* / pGP704-Tn*tgvAB*[N602A] | MFD*pir* carrying pGP704-Tn*tgvAB*[N602A]; Amp^R^, Gent^R^ | MB#11386 | This study |
| MFD*pir* / pGP704-TnCluster*-TgvAB*[H571A] | MFD*pir* carrying pGP704-TnCluster-*tgvAB*[H571A]; Amp^R^, Gent^R^ | MB#11901 | This study |
| MFD*pir* / pGP704-TnCluster*-hsdR*[K172A] | MFD*pir* carrying pGP704-TnCluster-*hsdR*[K172A]; Amp^R^, Gent^R^ | MB#11902 | This study |
| MG1655∆*araCBAD*-Tn*tgvA*[G94A]*B* | MG1655∆*araCBAD* with integrated Tn*tgvA*[G94A]*B*; Gent^R^ | MB#11389 | This study |
| MG1655∆*araCBAD*-Tn*tgvA*[R97A]*B* | MG1655∆*araCBAD* with integrated Tn*tgvA*[R97A]*B*; Gent^R^ | MB#11390 | This study |
| MG1655∆*araCBAD*-Tn*tgvAB*[G81A] | MG1655∆*araCBAD* with integrated Tn*tgvAB*[G81A]; Gent^R^ | MB#11387 | This study |
| MG1655∆*araCBAD*-Tn*tgvAB*[R84A] | MG1655∆*araCBAD* with integrated Tn*tgvAB*[R84A]; Gent^R^ | MB#11388 | This study |
| MG1655∆*araCBAD*-Tn*tgvAB*[H571A] | MG1655∆*araCBAD* with integrated Tn*tgvAB*[H571A]; Gent^R^ | MB#11391 | This study |
| MG1655∆*araCBAD*-Tn*tgvAB*[N593A] | MG1655∆*araCBAD* with integrated Tn*tgvAB*[N593A]; Gent^R^ | MB#11392 | This study |
| MG1655∆*araCBAD*-Tn*tgvAB*[N602A] | MG1655∆*araCBAD* with integrated Tn*tgvAB*[N602A]; Gent^R^ | MB#11393 | This study |
| MG1655∆*araCBAD-*TnCluster- *tgvAB[H571A]* | MG1655∆*araCBAD* with integrated TnCluster-*tgvAB*[*H571A*]; Gent^R^ | MB#11903 | This study |
| MG1655∆*araCBAD-*TnCluster-*HsdR*[K172A] | MG1655∆*araCBAD* with integrated TnCluster-*HsdR*[K172A]; Gent^R^ | MB#11904 | This study |
| TOP10 / P_[motif +]_  (pUC-Kan-mTn7T-Gm-*lacZ*) | TOP10 carrying plasmid P_[motif +]_; Kan^R^; Gent^R^ | MB#10698 | This study |
| TOP10 / P_[motif -]_ | TOP10 carrying plasmid P_[motif -]_; Kan^R^; Gent^R^ | MB#11378 | This study |
|  |  |  |  |
| **Plasmids** | | | |
| pBR-FLP | pBR322 derivative containing FLP+, λ cI857+, λ pR from pCP20 integrated into the EcoRV site of pBR322, used for FLP recombination; Amp^R^ | MB# 1203 | (13) |
| pUX-BF13 | pUX-BF13 - *ori*R6K, helper plasmid with Tn7 transposition function; Amp^R^ | MB#457  (S17-1λ*pir*) MB#4933  (MFD*pir*) | (14) |
| pGP704-Sac28 | pGP704-Sac28, *ori*R6K, *sacB*; Amp^R^ | MB#649 | (15) |
| p28-∆ddmABC | pGP704-Sac28 with ∆*ddmABC*; Amp^R^ | MB#9727 | This study |
| pGP704-TnAraC | pGP704-Tn*araC*; pGP704 with miniTn7 carrying *araC* and the *araBAD* promoter (*P*_BAD_); Amp^R^, Gent^R^ | MB#5513  (S17-1λpir) MB#8793  (MFD*pir*) | (3, 16) |
| pGP704-TnCluster | pGP704 with miniTn7 carrying *araC* and *P*_BAD_-*VC1769-65*; Amp^R^, Gent^R^ (TnCluster) | MB#11371 | This study |
| pGP704-TnT1RM | pGP704 with miniTn7 carrying *araC* and *P*_BAD_ -*VC1769-68-65*; Amp^R^, Gent^R^ (TnT1RM) | MB#11367 | This study |
| pGP704-Tn*tgvAB* | pGP704 with miniTn7 carrying *araC* and *P*_BAD_ -*VC1766-67*; Amp^R^, Gent^R^ (Tn*tgvAB*) | MB#11368 | This study |
| pGP704-Tn*tgvA* | pGP704 with miniTn7 carrying *araC* and *P*_BAD_ -*VC1767*; Amp^R^, Gent^R^ (Tn*tgvA*) | MB#11369 | This study |
| pGP704-Tn*tgvB* | pGP704 with miniTn7 carrying *araC* and *P*_BAD_ -*VC1766*; Amp^R^, Gent^R^ (Tn*tgvB*) | MB#11370 | This study |
| pGP704-Tn*tgvA*[G94A]*B* | pGP704 with miniTn7 carrying *araC* and *P_BAD_* -*VC1766-67* encoding G94A variant of *VC1767*; Amp^R^, Gent^R^ (Tn*tgvA*[G94A]B) | MB#11382 | This study |
| pGP704-Tn*tgvA*[R97A]*B* | pGP704 with miniTn7 carrying *araC* and *P*_BAD_ -*VC1766-67* encoding R97A variant of *VC1767*; Amp^R^, Gent^R^ (Tn*tgvA*[R84A]B) | MB#11383 | This study |
| pGP704-Tn*tgvAB*[G81A] | pGP704 with miniTn7 carrying *araC* and *P_BAD_* -*VC1766-67* encoding G81A variant of *VC1766*; Amp^R^, Gent^R^ (Tn*tgvAB*[G81A]) | MB#11380 | This study |
| pGP704-Tn*tgvAB*[R84A] | pGP704 with miniTn7 carrying *araC* and *P_BAD_* -*VC1766-67* encoding R84A variant of *VC1766*; Amp^R^, Gent^R^ (Tn*tgvAB*[R84A]) | MB#11381 | This study |
| pGP704-Tn*tgvAB*[H571A] | pGP704 with miniTn7 carrying *araC* and *P_BAD_* -*VC1766-67* encoding H571A variant of *VC1766*; Amp^R^, Gent^R^ (Tn*tgvAB*[H571A]) | MB#11384 | This study |
| pGP704-Tn*tgvAB*[N593A] | pGP704 with miniTn7 carrying *araC* and *P*_BAD_ -*VC1766-67* encoding N593A variant of *VC1766*; Amp^R^, Gent^R^ (Tn*tgvAB*[N593A]) | MB#11385 | This study |
| pGP704-Tn*tgvAB*[N602A] | pGP704 with miniTn7 carrying *araC* and *P*_BAD_ -*VC1766-67* encoding N602A variant of *VC1766*; Amp^R^, Gent^R^ (Tn*tgvAB*[N602A]) | MB#11386 | This study |
| pUC18-miniTn7T-Gm-*lacZ* | pUC18-miniTn7T-Gm-*lacZ*; Amp^R^, Gent^R^ | MB#10656 | Addgene # 63120 (17) |
| P_[motif +]_ | pUC-Kan-mTn7T-Gm-*lacZ* derived from pUC18-miniTn7T-Gm-*lacZ* by replacing *bla* (Amp^R^) by *aph* (Kan^R^) cassette; harboring motif [GATGCCCATACTT] within *aacC1* (Gent^R^)*;* Kan^R^, Gent^R^ | MB#10698 | This study |
| P_[motif -]_ | pUC-Kan-mTn7T-Gm-*lacZ*, harboring silent mutations within *aacC1* (Gent^R^) to modify motif [**A**AT**A**CCCAT**TGAA**]; Kan^R^, Gent^R^ | MB#11378 | This study |

^a^ *V. cholerae* locus tag numbers are according to Heidelberg *et al*.(18)

^b^ Kan – Kanamycin; Rif – Rifampicin; Strep – Streptomycin; Gent – Gentamicin; Amp – Ampicillin

**Reference:**

1. Matthey N, Drebes Dörr NC, Blokesch M. 2018. Long-Read-Based Genome Sequences of Pandemic and Environmental *Vibrio cholerae* Strains. Microbiol Resour Announc 7:e01574-18.

2. Yildiz FH, Schoolnik GK. 1998. Role of *rpoS* in Stress Survival and Virulence of *Vibrio cholerae*. J Bacteriol 180:773–784.

3. Stutzmann S, Blokesch M. 2020. Comparison of chitin‐induced natural transformation in pandemic *Vibrio cholerae* O1 El Tor strains. Environ Microbiol 22:4149–4166.

4. Wachsmuth IK, Evins GM, Fields PI, Olsvik O, Popovic T, Bopp CA, Wells JG, Carrillo C, Blake PA. 1993. The Molecular Epidemiology of Cholera in Latin America. J Infect Dis 167:621–626.

5. Pearson GDN, Woods A, Chiang SL, Mekalanos JJ. CTX genetic element encodes a site-specific recombination system and an intestinal colonization factor. Proc. Natl. Acad. Sci. USA 90:3750-3754.

6. Miller VL, DiRita VJ, Mekalanos JJ. 1989. Identification of *toxS*, a regulatory gene whose product enhances ToxR-mediated activation of the cholera toxin promoter. J Bacteriol 171:1288–1293.

7. Borgeaud S, Metzger LC, Scrignari T, Blokesch M. The type VI secretion system of *Vibrio cholerae* fosters horizontal gene transfer. Science 347:63-67.

8. Waldor MK, Mekalanos JJ. 1996. Lysogenic Conversion by a Filamentous Phage Encoding Cholera Toxin. Science 272:1910–1914.

9. Jaskólska M, Adams DW, Blokesch M. 2022. Two defence systems eliminate plasmids from seventh pandemic *Vibrio cholerae*. Nature 604:323–329.

10. Simon R, Priefer U, Pühler A. 1983. A Broad Host Range Mobilization System for *In Vivo* Genetic Engineering: Transposon Mutagenesis in Gram Negative Bacteria. Bio/Technology 1:784–791.

11. Ferrières L, Hémery G, Nham T, Guérout A-M, Mazel D, Beloin C, Ghigo J-M. 2010. Silent Mischief: Bacteriophage Mu Insertions Contaminate Products of *Escherichia coli* Random Mutagenesis Performed Using Suicidal Transposon Delivery Plasmids Mobilized by Broad-Host-Range RP4 Conjugative Machinery. J Bacteriol 192:6418–6427.

12. Aoki SK, Lillacci G, Gupta A, Baumschlager A, Schweingruber D, Khammash M. 2019. A universal biomolecular integral feedback controller for robust perfect adaptation. Nature 570:533–537.

13. De Souza Silva O, Blokesch M. 2010. Genetic manipulation of *Vibrio cholerae* by combining natural transformation with FLP recombination. Plasmid 64:186–195.

14. Bao Y, Lies DP, Fu H, Roberts GP. 1991. An improved Tn7-based system for the single-copy insertion of cloned genes into chromosomes of gram-negative bacteria. Gene 109:167–168.

15. Meibom KL, Li XB, Nielsen AT, Wu C-Y, Roseman S, Schoolnik GK. 2004. The *Vibrio cholerae* chitin utilization program. Proc Natl Acad Sci 101:2524–2529.

16. Adams DW, Stutzmann S, Stoudmann C, Blokesch M. 2019. DNA-uptake pili of *Vibrio cholerae* are required for chitin colonization and capable of kin recognition via sequence-specific self-interaction. Nat Microbiol 4:1545–1557.

17. Choi K-H, Gaynor JB, White KG, Lopez C, Bosio CM, Karkhoff-Schweizer RR, Schweizer HP. 2005. A Tn7-based broad-range bacterial cloning and expression system. Nat Methods 2:443–448.

18. Heidelberg JF, Eisen JA, Nelson WC, Clayton RA, Gwinn ML, Dodson RJ, Haft DH, Hickey EK, Peterson JD, Umayam L, Gill SR, Nelson KE, Read TD, Tettelin H, Richardson D, Ermolaeva MD, Vamathevan J, Bass S, Qin H, Dragoi I, Sellers P, McDonald L, Utterback T, Fleishmann RD, Nierman WC, White O, Salzberg SL, Smith HO, Colwell RR, Mekalanos JJ, Venter JC, Fraser CM. 2000. DNA sequence of both chromosomes of the cholera pathogen *Vibrio cholerae*. Nature 406:477–483.
